# FMRP ligand circZNF609 destabilizes RAC1 mRNA to reduce metastasis in acral melanoma and cutaneous melanoma

**DOI:** 10.1186/s13046-022-02357-7

**Published:** 2022-05-10

**Authors:** Qingfeng Shang, Haizhen Du, Xiaowen Wu, Qian Guo, Fenghao Zhang, Ziqi Gong, Tao Jiao, Jun Guo, Yan Kong

**Affiliations:** Key Laboratory of Carcinogenesis and Translational Research (Ministry Education), Department of Melanoma and Sarcoma, Peking University Cancer Hospital and Research Institute, Beijing, China

**Keywords:** circZNF609, Acral melanoma, Cutaneous melanoma, FMRP, RAC1

## Abstract

**Background:**

Melanoma is a type of malignant tumor with high aggressiveness and poor prognosis. At present, metastasis of melanoma is still an important cause of death in melanoma patients. However, the potential functions and molecular mechanisms of most circular RNAs (circRNAs) in melanoma metastasis remain unknown.

**Methods:**

circRNAs dysregulated in melanoma cell subgroups with different metastatic abilities according to a screening model based on repeated Transwell assays were identified with a circRNA array. The expression and prognostic significance of circZNF609 in skin cutaneous melanoma and acral melanoma cells and tissues were determined by qRT–PCR, nucleoplasmic separation assays and fluorescence in situ hybridization. In vitro wound healing, Transwell and 3D invasion assays were used to analyse melanoma cell metastasis ability. Tail vein injection and intrasplenic injection were used to study in vivo lung metastasis and liver metastasis, respectively. The mechanism of circZNF609 was further evaluated via RNA immunoprecipitation, RNA pull-down, silver staining, and immunofluorescence colocalization assays.

**Results:**

circZNF609 was stably expressed at low levels in melanoma tissues and cells and was negatively correlated with Breslow depth, clinical stage and prognosis of melanoma patients. circZNF609 inhibited metastasis of acral and cutaneous melanoma in vivo and in vitro. Mechanistically, circZNF609 promoted the binding of FMRP protein and RAC1 mRNA, thereby enhancing the inhibitory effect of FMRP protein on the stability of RAC1 mRNA and ultimately inhibiting melanoma metastasis.

**Conclusions:**

Our findings revealed that circZNF609 plays a vital role in the metastasis of acral and cutaneous melanoma through the circRNF609-FMRP-RAC1 axis and indicated that circZNF609 regulates the stability of RAC1 mRNA by combining with FMRP, which might provide insight into melanoma pathogenesis and a new potential target for treatment of melanoma.

**Supplementary Information:**

The online version contains supplementary material available at 10.1186/s13046-022-02357-7.

## Background

Melanomas are thought to originate from the neuroectoderm and are characterized by high malignancy and poor prognosis. Although early-stage melanoma can be treated by surgical resection, melanoma has a strong tendency to disseminate and metastasize, and it easily invades many vital organs, such as the liver, brain, and lung, through local invasion and even lymphatic metastasis and blood metastasis. According to previous studies, 44% of patients with metastatic melanoma will develop symptomatic brain metastases, and metastatic melanoma cells are usually resistant to drugs and have poor sensitivity to radiotherapy and chemotherapy [1–3]. Therefore, melanoma metastasis is still currently an important cause of death in melanoma patients. On the other hand, there are huge differences between Asian melanoma and Caucasian melanoma in terms of primary site, clinical manifestations, genomics and treatment sensitivity. Caucasian melanomas are predominantly located in the skin of non-acral sites, and most are superficial spreading melanoma histologically.

While Asian melanomas are mainly of the acral lentiginous melanoma, due to their preferential occurrence in the skin of the acral sites, which progress faster and have a worse prognosis [4–6]. Therefore, exploring how melanomas, especially specific types of melanomas other than common types, can metastasize to multiple organs in a variety of ways and identifying a reliable target that can inhibit the metastatic ability of melanoma cells are major challenges in melanoma research.

Circular RNAs (circRNAs) are covalently closed, single-stranded circular transcripts without a 5′ cap or a 3′ poly(A) tail; this structural characteristic gives circRNAs a longer half-life than linear mRNAs [7]. In addition, some circRNAs are evolutionarily conserved across species, with cell-specific and tissue-specific characteristics [8]. With the continuous advancements in sequencing technology, an increasing number of circRNAs have been discovered. At the same time, several research groups have discovered that circRNAs not only participate in the regulation of a variety of physiological processes, such as haematopoietic stem cell differentiation [9], neural differentiation [10], and innate immunity [11] but also participate in many aspects of tumorigenesis. For example, circHIPK3 regulates the progression of a variety of tumors by binding to diverse miRNAs [12]. However, circRNAs, especially those that play critical roles in specific types of melanoma, are rarely studied in melanoma.

Fragile X mental retardation protein (FMRP or FMR1) is an RNA binding protein (RBP) that is highly expressed in neurons [13]. Previous studies have claimed that FMRP can participate in RNA stability regulation [14] and the DNA damage process [15] and act as a translation regulator [16–18]. Initially, the relationship between FMRP and cancer was identified due to the observation that patients with Fragile X Syndrome (FXS) have a decreased risk of cancer [19]. Subsequently, studies revealed that FMRP is overexpressed in hepatocellular carcinoma and breast cancer and participates in regulation of the tumor metastasis phenotype [20, 21]. RAS-related C3 botulinum toxin substrate 1 (RAC1) is a core member of the Rho subfamily in the Ras superfamily and is involved in the biological processes of cell migration, invasion and adhesion [22]. Researchers have revealed that FMRP may regulate the translation process of RAC1 in Drosophila and mice [23, 24], but the relationship between the two in melanoma is unknown.

In this study, we identified circZNF609 as a downregulated circRNA in melanoma. The interaction of circZNF609 and FMRP protein can promote the binding of FMRP to the mRNA of its downstream target RAC1, thereby enhancing the inhibitory effect of FMRP on the stability of RAC1 mRNA, ultimately downregulating the RAC1 mRNA and protein levels and inhibiting the metastasis of melanoma cells.

## Materials and methods

### Cell culture

The detailed information of human cutaneous melanoma cell lines and acral melanoma cells, growth media, and their metastatic state are in Table S1. All cells were grown in a humidified incubator at 37 °C with 5% CO2 and authenticated by STR analysis. Cells were maintained in culture for no more than 20 passages, excluding passaging prior to receipt in our lab. All cell lines were routinely tested negative for mycoplasma contamination.

### Clinical sample collection

Human melanoma samples were obtained with informed consent, and the use of clinical samples was approved by the Medical Ethics Committee of the Beijing Cancer Hospital & Institute. All samples were diagnosed via histopathological examination, including haematoxylin and eosin (H&E) staining and immunohistochemistry (IHC) for melanoma markers (S-100, MART-1 and HMB-45).

### Isolation of highly invasive HMY-1 cell sublines

Transwell chambers with 8 μm pores in 6-well plates (Corning, USA) were used to obtain highly invasive HMY-1 cell sublines. First, 7 × 10^5^ HMY-1 cells in 1 mL serum-free DMEM were seeded into the upper chamber, which was precoated with Matrigel (BD Biosciences, USA), and 2 mL of DMEM supplemented with 25% foetal bovine serum was placed into the lower well. After incubation for 48 h, the invasive cells on the bottom of the chambers were harvested. Then, the harvested invasive cells were cultured and filtered for another 6 rounds using the Transwell chambers. After 7 continuous invasion assays, a highly invasive HMY-1 subline derived from the HMY-1 cell line was obtained. The invasive ability of these two cell lines was then confirmed by Transwell assays and lung metastasis models.

### Purification of highly invasive HMY-1 clones

Five hundred highly invasive HMY-1 cells were evenly seeded onto a 10-cm dish. When a single cell grew to a visible cell cluster, a sterilized clone ring was used to get the clones. Different clones were separately cultured in a 24-well plate and passed on to 6-well plate when the plate was full.

### RNA extraction and qRT–PCR

Total RNA was extracted from FFPE samples and cell lines using an RNeasy FFPE Kit (Qiagen, Germany) and TRIzol reagent (Life Technologies, USA), respectively. Then, cDNA was synthesized using a PrimeScript RT reagent kit (Takara, Japan) according to the manufacturer’s instructions. Quantitative reverse transcription polymerase chain reaction (qRT–PCR) was performed using SYBR Green Real-time PCR Master Mix (TOYOBO, Japan). The sequence for each primer is listed in Table S2.

### circRNA sequencing and microarray assay

For circRNA sequencing, total RNA was isolated from HMY-1 cells and highly invasive HMY-1 cells using TRIzol reagent (Life Technologies, USA) and purified with an RNease Mini kit (Qiagen, Germany). Then, circRNAs were sequenced using a human ceRNA Array V3.0 (4x180K, designed by Shanghai Biotechnology Corporation, and made by Agilent Technologies), which contains 105,509 circRNA probes.

### Actinomycin D and RNase R treatment

Actinomycin D and RNase R treatment experiments were performed to assess the stability of circZNF609 and ZNF609 mRNA. In brief, cells were treated with 2 mg/ml actinomycin D in a 6-well plate. After incubation for several hours, the cells were collected. For RNase R treatment, total RNA (2 μg) was incubated for 15 min at 37 °C with 3 U/μg RNase R (Epicentre, WI, USA). After treatment with actinomycin D or RNase R, the circZNF609, ZNF609 and RAC1 mRNA expression was measured via qRT–PCR.

### Small interfering RNA (siRNA) and transfection

siRNA duplexes for knockdown of circZNF609, ZNF609 and both were synthesized by GenePharma (Suzhou, China). Cells were transfected with the siRNAs using Lipofectamine RNAiMAX (Invitrogen, USA) according to the manufacturer’s instructions. The siRNA sequences are listed in Table S2.

### circRNA plasmid construction and stable transfection

The circZNF609 overexpression plasmid and vector plasmid were obtained from Geneseed (Guangzhou, China). 293 T cells were transfected with the plasmids to package the lentivirus using Lipofectamine 3000 (Invitrogen, USA) according to the manufacturer’s instructions. Human melanoma cells were infected with the packaged lentivirus and selected with 0.5–2 μg/ml puromycin for 4 days. Surviving melanoma cells exhibiting GFP expression were then selected for subsequent culture.

### Nucleoplasmic separation assay

The nuclear and cytoplasmic fractions were extracted using a Minute™ Cytoplasmic and Nuclear Extraction Kit (Invent Biotechnologies, USA) according to the manufacturer’s instructions. In brief, cells were washed with cold PBS for 2 min. Then, cell lysis buffer was added to the cells on ice. After incubation for 5 min, the cell lysate was transferred to a prechilled 1.5 ml microcentrifuge tube. The tube was vigorously vortexed for 15 s. After centrifugation of the tube for 5 min at top speed at 4 °C, the supernatant was collected as the cytosolic fraction. Then, an appropriate amount of nuclear extraction buffer was added to the pellet, and the tube was vigorously vortexed for 15 s and incubated on ice for 1 min. The 15 s of vortexing and 1 min incubation were repeated 4 times. After that, the nuclear fraction was transferred to a prechilled filter cartridge with a collection tube and centrifuged at top speed in a microcentrifuge for 30 s.

### Fluorescence in situ hybridization (FISH)

A Cy3-labelled specific probe for circZNF609 was designed and synthesized by RiboBio. The FISH experiment was performed using a FISH kit (RiboBio, China) according to the manufacturer’s instructions.

### Immunofluorescence (IF)

Human melanoma cells were fixed with 4% paraformaldehyde for 15 min and then blocked with 5% BSA for 30 min. The melanoma cells were incubated with primary antibodies at 4 °C overnight and fluorescence-conjugated secondary antibodies at room temperature for 1 h. After washing with PBS, the cells were counterstained with DAPI for 10 min and imaged under a microscope (Olympus Corp).

### Wound healing assays

Wound healing assays were performed using Culture–Insert (Ibidi, Germany) according to the manufacturer’s instructions. In brief, 1 × 10^5^ human melanoma cells in 70 μl complete medium were seeded into each culture well and incubated for 24 h, and then, the culture insert was gently removed using sterile tweezers. After washing with PBS, the cells were cultured in culture medium with 1% FBS. Images were taken at several time points.

### Transwell assays

Human melanoma cells were cultured in serum-free culture medium 24 h before performing Transwell assays. Cell culture medium supplemented with 25% foetal bovine serum was added to the bottom chambers of a 24-well plate. For the invasion and migration assays, 1 × 10^5^ melanoma cells in serum-free medium were seeded into the top chamber with or without Matrigel (BD Biosciences, USA), respectively. After incubation for several hours, the Transwell inserts were fixed in 4% paraformaldehyde for 10 min and then stained with 1% crystal violet for 10 min. Cells that did not migrate through the 8 μm pores of the Transwell chambers were removed using cotton swabs. Migratory or invasive melanoma cells located on the bottom of the chamber were counted using an inverted phase-contrast microscope.

### 3D spheroid-based Matrigel invasion assay

3D spheroid-based Matrigel invasion assays were performed as previously described [25–27]. In brief, 1 × 10^4^ LM-MEL-45 cells in 200 μl complete culture medium were grown in ultralow attachment (ULA) 96-well round-bottom plates (Corning, USA) for 3 days. After tumor spheroids formed, 100 μl of growth medium was gently removed from the spheroid plates. Then, 100 μl Matrigel (BD Biosciences, USA) was gently dispensed into the U-bottom well. After incubation in an incubator at 37 °C for 1 h, 100 μl complete growth medium was gently added to each well. Three days later, images of invasive tumor spheroids were taken with an inverted phase-contrast microscope.

### Animal study

All animal experiments were approved by the Medical Ethics Committee of the Beijing Cancer Hospital & Institute. For in vivo cutaneous melanoma lung metastasis assays, 4-week-old female BALB/c nude mice were intravenously injected with 5 × 10^5^ A2058 cells in 100 μl PBS via the tail vein. The mice were sacrificed when they became weak, hunched and emaciated. For in vivo acral melanoma lung metastasis assays, 5-week-old female NOD/SCID mice were intravenously injected with 2 × 10^6^ HMY-1 cells in 200 μl PBS via the tail vein. The mice were sacrificed when they became weak, hunched and emaciated. For in vivo acral melanoma liver metastasis assays, 1 × 10^6^ HMY-1 cells suspended in 50 μl PBS were injected into the inferior hemispleen of 4-week-old female NOG mice. The mice were euthanized after 4 weeks. The lungs and livers were excised, and then, the number of metastatic tumor nodules in the lungs and livers was carefully counted. Subsequently, all the samples were fixed with 4% paraformaldehyde and analysed via H&E staining. To detect tumors in live animals, mice were injected with 100 μl D-luciferin (15 mg/ml) and then anaesthetized with isoflurane. Ten minutes later, bioluminescence images were acquired and analysed using an IVIS Spectrum In Vivo Imager.

### RNA pull-down assay

The biotin-labelled circZNF609 probe was synthesized by RiboBio, and the RNA pull-down assay was performed using a Pierce™ Magnetic RNA-Protein Pull-Down Kit (Thermo Fisher, USA) according to the manufacturer’s instructions. First, the biotin-labelled circZNF609 probe or control probe was incubated with streptavidin magnetic beads at room temperature for 30 min. Then, the melanoma cell lysates were incubated with probe-bead complexes at 4 °C for 1 h to allow binding of RBPs to RNAs. Subsequently, the RNA-protein complexes were washed and eluted from beads via incubation for 30 min at 37 °C with agitation. The eluted proteins were finally analysed via silver staining and Western blotting. The circZNF609 probe and control probe sequences used are listed in Table S2.

### Silver staining

Silver staining was performed using a Fast Silver Stain Kit (Beyotime, China) according to the manufacturer’s instructions.

### RNA immunoprecipitation (RIP)

RIP assays were performed using a Magna RIP RNA-Binding Protein Immunoprecipitation Kit (Millipore) as described in the protocol. Briefly, human melanoma cells were lysed in RIP lysis buffer on ice for 5 min. After washing, 50 μl magnetic beads were incubated with 5 μg antibodies with rotation for 30 min at room temperature. After incubation, the melanoma cell lysates were incubated with bead-antibody complexes at 4 °C overnight. Subsequently, the immune complexes were washed six times with washing buffer, and the remaining RNA was purified and extracted for later qRT–PCR analysis and RIP sequencing.

### Statistical analysis

All experiments were carried out at least three times, and the results are presented as the mean ± standard deviation (S.D.). Statistical analysis was performed using Student’s two-tailed t test or one-way analysis of variance (ANOVA). The expression correlations in melanoma samples were assessed using Pearson correlation analysis. Survival curves were assessed using the Kaplan–Meier method and compared with log-rank tests. Statistical significance was defined as a *P* value of less than 0.05 (*P < 0.05, **P < 0.01, ***P < 0.001); ns: not significant (*P* > 0.05).

## Results

### Identification and characteristics of circZNF609 in melanoma cells

**Fig. 1:**
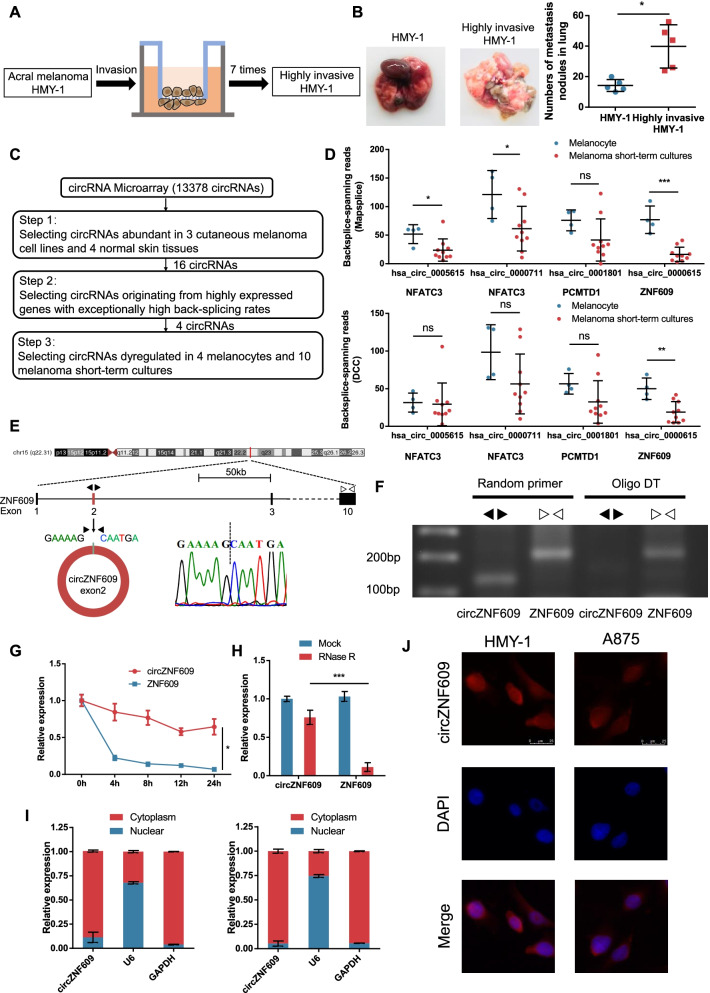
The identification and characteristics of circZNF609 in human melanoma cells. **a** The sketch map of cell invasion model. **b** In vivo lung metastasis model to verify the different metastatic potential between HMY-1 and highly invasive HMY-1 sublines. **c** Flowchart illustrating the screening criteria of potential regulatory circRNAs in acral melanoma and cutaneous melanoma. **d** The expression of 4 circRNAs in 4 melanocytes and 10 melanoma short-term cultures of GSE138711 according to Mapsplice algorithm (top) and DCC algorithm (bottom). **e** The formation of circZNF609. circZNF609 derived from back-spliced exons 2 of genomic ZNF609. The existence of circZNF609 was detected by Sanger sequencing. Black arrows represent divergent primers and white arrows represent convergent primers. **f** PCR products with different primers showing circularization of circZNF609. Random primers amplified circZNF609 in cDNA. Oligo DT did not amplify circZNF609 in cDNA. Linear ZNF609 was used as a control. **g** qRT–PCR analysis for the expression of circZNF609 and ZNF609 mRNAs after treatment with Actinomycin D in HMY-1 cells. **h** qRT–PCR analysis for the expression of circZNF609 and ZNF609 mRNAs after treatment with RNase R in HMY-1 cells. **i** Nucleoplasmic separation assay showing that circZNF609 mainly located in the cytoplasm in HMY-1 (left) and A875 cells (right). GAPDH and U6 were applied as positive controls in the cytoplasm and nucleus, respectively. **j** FISH assays showing the predominant cytoplasmic distribution of circZNF609 in HMY-1 and A875 cells

To investigate the key driver molecule in the metastasis of melanoma, we first applied cell invasion models to mimic the invasion process in vitro (Fig. 1a). After 7 continuous selections, a highly invasive HMY-1 subline was established, and metastatic ability was further verified by in vitro Transwell assays (Fig. S1a-c) and in vivo lung metastasis experiments (Fig. 1c). Subsequently, we conducted circRNA transcriptome analysis of HMY-1 cells and the highly invasive HMY-1 cells using a human ceRNA array. A total of 13,378 dysregulated circRNAs were identified in these cells harbouring different metastatic abilities. Considering that circRNAs are tissue-specific and cell-specific and that most circRNAs may result from splicing errors [8, 28], we conducted further screening to select high-abundance circRNAs that truly participate in the progression of melanoma (Fig. 1c). Among these circRNAs, we identified 4 dysregulated circRNAs that met the following requirements (Fig. S2d, Table S2): (1) fold change > 1.3 or < 0.7 in the array; (2) detected in 3 cutaneous melanoma cell lines and 4 normal skin tissues [29] (Fig. S2a-c); and (3) circRNAs originating from highly expressed genes with exceptionally high back-splicing rates [28]. Next, we analysed the expression of 4 circular RNAs in 4 melanocytes and 10 cutaneous melanoma short-term cultures from GSE138711 [30]. We found that only circZNF609 was downregulated in cutaneous melanoma, which was consistent with the trend in acral melanoma (Fig. 1d). Therefore, these findings led us to study circZNF609 in acral melanoma and cutaneous melanoma.

According to the CircBase database [31], circZNF609 is an exonic circRNA that is formed by exon 2 of the ZNF609 gene. Then, we verified the existence and circular characteristics of circZNF609 through several experiments. The back-spliced junction of circZNF609 was amplified using divergent primers and confirmed by Sanger sequencing (Fig. 1e). Since circRNAs are single-stranded circular transcripts without a 3′ poly(A) tail and oligo dT can only amplify linear transcripts containing a poly(A) tail, circZNF609 could not be amplified when only oligo dT and divergent primers were used (Fig. 1f). We also observed that circZNF609 had a longer half-life than ZNF609 mRNA (Fig. 1g) and was resistant to digestion with RNase R (Fig. 1h), further demonstrating its circular characteristic. In addition, using a nucleoplasmic separation assay and FISH, we confirmed that circZNF609 was predominantly localized in the cytoplasm of melanoma cells (Fig. 1i, j). Taken together, these results indicate that circZNF609 is an abundant, stable, cytoplasmic and exonic circRNA in human melanoma cells.

### circZNF609 is downregulated in melanoma and correlated with melanoma progression and prognosis

**Fig. 2:**
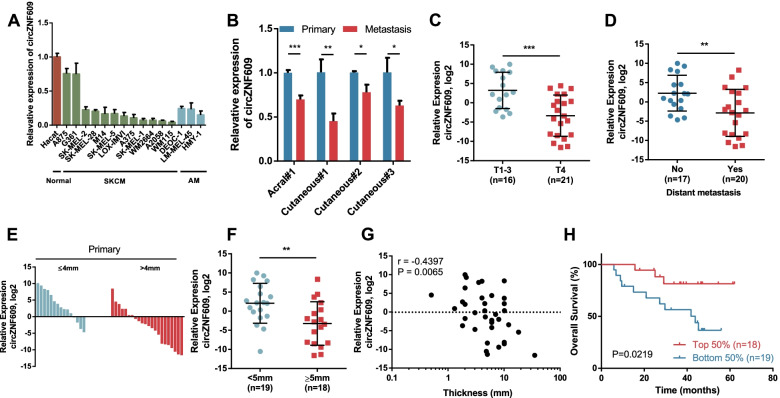
The expression and clinical significance of circZNF609 in melanoma. **a** Detection of circZNF609 in human normal cell line and melanoma cell lines by qRT–PCR. **b** Detection of circZNF609 in primary melanoma tissue and paired melanoma metastasis by qRT–PCR. **c-f** The expression levels of circZNF609 in cutaneous melanoma patients with different pathological T stages, metastasis state and Breslow depth (*n* = 37). **g** Correlation of circZNF609 expression from primary cutaneous melanoma patient samples with Breslow thickness (*n* = 37). **h** Kaplan-Meier survival analysis of cutaneous melanoma patients according to the expression level of circZNF609 (*n* = 37)

Next, we examined the expression of circZNF609 in human melanoma cell lines. qRT–PCR revealed that circZNF609 was downregulated in 12 cutaneous melanoma cell lines and 3 acral melanoma cell lines compared with the normal keratinocyte cell line HaCaT (Fig. 2a). Besides, circZNF609 was downregulated in highly invasive HMY-1, which was consistent with the results of circRNA sequencing (Fig. S1d). Moreover, we found that circZNF609 was also downregulated in metastatic tissue than that in paired primary melanoma tissues (Fig. 2b). We further investigated the clinical significance of circZNF609 in cutaneous melanoma patients from our hospital. In a cohort of 37 FFPE primary cutaneous melanoma samples, we observed progressive loss of circZNF609 expression (Fig. 2b). Cutaneous melanoma patients with distant metastasis and deeper Breslow depth had lower circZNF609 expression (Fig. 2c-e). Intriguingly, we also found that the abundance of circZNF609 was significantly negatively correlated with the depth of melanoma invasion (Fig. 2f). Meanwhile, a low abundance of circZNF609 was also associated with shorter overall survival (OS) (Fig. 2g). Furthermore, correlation analysis demonstrated that low expression of circZNF609 was positively correlated with aggressive clinicopathological.

characteristics, especially distant metastasis and Breslow depth (Table 1). Collectively, these data show that circZNF609 was downregulated in cutaneous melanoma and acral melanoma, and its abundance was associated with cutaneous melanoma progression and patient prognosis, indicating that circZNF609 expression might have prognostic value for melanoma patients.

**Table 1:** Association between circZNF609 expression and clinical features of cutaneous melanoma

			circZNF609 expression		
Characteristics		Number of cases	Low(*n* = 19)	High(*n* = 18)	*P*-value(χ^2^ test)
Gender	Male	23	11	12	0.5824
	Female	14	8	6	
Age	< 50	19	10	9	0.8728
	≥50	18	9	9	
Tumor location	Upper region	29	16	13	0.376
	Lower region	8	3	5	
Pathology stage	T1–3	16	5	11	**0.0327**
	T4	21	14	7	
Breslow depth	< 5 mm	19	6	13	**0.0134**
	≥5 mm	18	13	5	
Ulceration	Yes	15	9	6	0.3848
	No	22	10	12	
Distant metastasis	Yes	20	14	6	**0.0138**
	No	17	5	12	

### circZNF609 inhibits melanoma cell metastasis in vitro

**Fig. 3:**
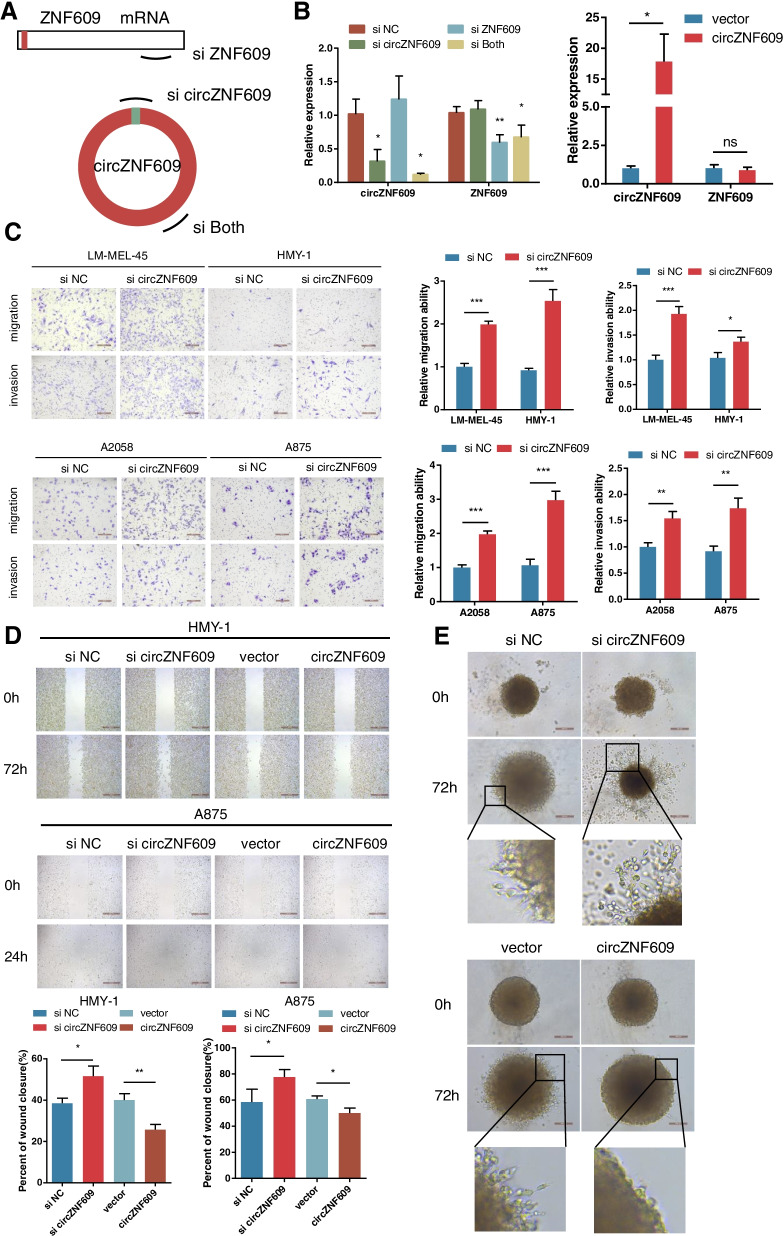
circZNF609 depletion drives melanoma metastasis in vitro. **a** Schematic illustration showing three targeted siRNAs. **b** Left, qRT-PCR analysis of circZNF609 and ZNF609 mRNA in LM-MEL-45 cells treated with siRNAs. Right, circZNF609 overexpression efficiency in LM-MEL-45 cells. **c** Cell migratory and invasive capabilities were assessed by Transwell assay after knocking down of circZNF609 in 2 acral melanoma cells and 2 cutaneous melanoma cells. **d** Cell migratory capability was assessed by wound healing assay after knocking down or overexpressing circZNF609 in 2 melanoma cells. **e** Cell invasive capability was assessed by 3D spheroid-based Matrigel invasion assay after knocking down or overexpressing circZNF609 in LM-MEL-45 cells

We verified that circZNF609 was downregulated in the highly invasive HMY-1 subline and that its abundance was negatively correlated with the depth of melanoma invasion and distant metastasis state. These results suggest that circZNF609 loss might contribute to melanoma progression, especially melanoma metastasis. To test whether circZNF609 can affect melanoma metastasis, loss-of-function and gain-of-function assays were performed. We first designed 3 siRNAs: one targeting the back-splice region of circZNF609, another targeting a sequence only in ZNF609 mRNA and a third targeting a sequence present in both the linear and circular transcripts (Fig. 3a). After transfection of LM-MEL-45 cells with the siRNAs for 48 h, the cells were harvested, and the expression of circZNF609 and ZNF609 was analysed via qRT–PCR (Fig. 3b). As expected, siRNA directed against the back-splice region only knocked down circZNF609 and did not affect the expression of ZNF609 mRNA (Fig. S3a-c). siRNA targeting sequences present in both the linear and circular transcripts effectively knocked down both transcripts. Simultaneously, circZNF609-overexpressing cells were successfully constructed, and no significant change in ZNF609 mRNA was observed (Fig. 3b, Fig. S3a-c).

To investigate the impact of circZNF609 on the metastatic phenotypes of melanoma cells, we selected 2 cutaneous melanoma cell lines (A2058 and A875) and 2 acral melanoma cell lines (HMY-1 and LM-MEL-45); the A875 and LM-MEL-45 lines retained high basal circZNF609 expression, and the A2058 and HMY-1 lines exhibited low basal circZNF609 expression. Transwell assays and wound healing assays showed that the migration and invasion capacities of the 2 cutaneous melanoma cell lines and 2 acral melanoma cell lines were significantly enhanced in response to circZNF609 silencing (Fig. 3c, d, Fig. S4c). Similarly, ectopic expression of circZNF609 in the 4 melanoma cell lines led to a significant decrease in cell migration and invasion abilities (Fig. 3d, Fig. S4a, b). Moreover, in 3D invasion assays, we observed that circZNF609 loss in LM-MEL-45 cells resulted in an enhanced invasion area and an increased number of protrusions (Fig. 3e). Conversely, stable circZNF609 overexpression remarkably reduced the invasion area and the number of protrusions (Fig. 3e). Taken together, these findings suggest that circZNF609 modulation in cell models altered cutaneous melanoma and acral melanoma migration and invasion in vitro.

### circZNF609 suppresses melanoma metastasis in vivo

**Fig. 4:**
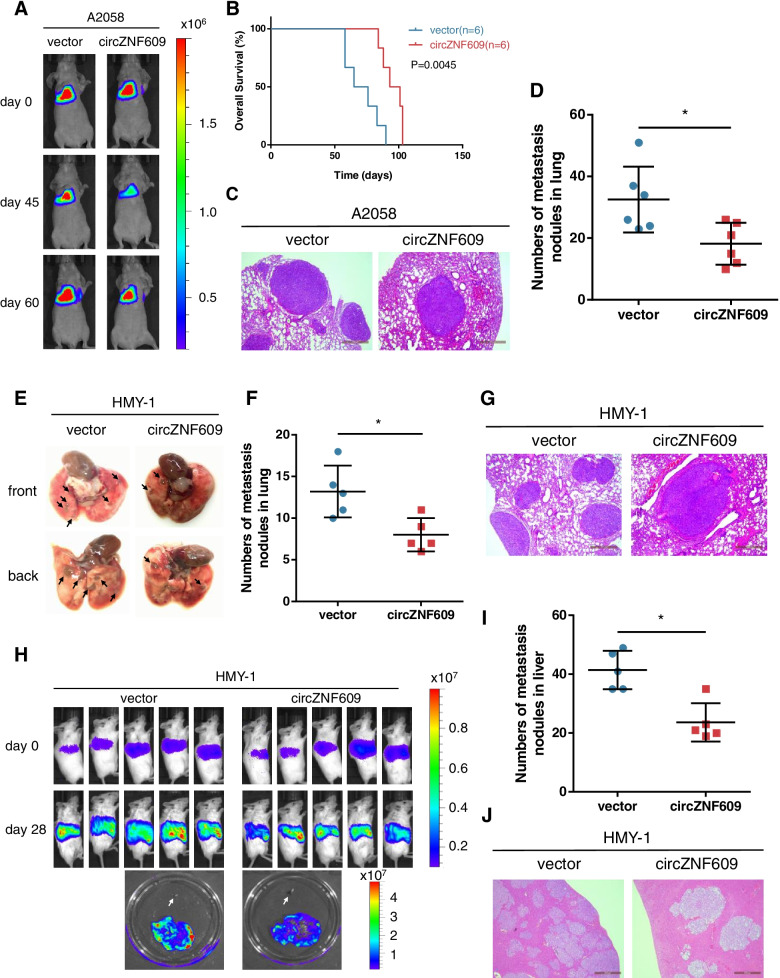
Ectopic expression of circZNF609 suppresses melanoma metastasis in vivo. **a-d** Representative bioluminescent images (**a**), Kaplan-Meier survival analysis (**b**), H&E staining (**c**) and quantification (**d**) of lung metastatic colonization of nude mice treated with tail-vein injection of firefly luciferase-expressing A2058 cells stably transfected with vector or circZNF609. **e-g** Representative images (**e**), quantification (**f**) and H&E staining (**g**) of lung metastatic colonization of NOD/SCID mice treated with tail-vein injection of HMY-1 cells stably transfected with vector or circZNF609. **h-j** Representative bioluminescent images (**h**), quantification (**i**) and H&E staining (**j**) of liver metastatic colonization of NOG mice treated with tail-vein injection of firefly luciferase-expressing HMY-1 cells stably transfected with vector or circZNF609. White arrows represent hemispleen

To identify the effect of circZNF609 on melanoma metastasis in vivo, we established lung and liver metastasis models by implanting melanoma cells transfected with circZNF609 or vector. Firefly luciferase-expressing A2058 cells transfected with circZNF609 or vector were inoculated into female nude mice via tail vein injection. The mice were further monitored and analysed for lung metastasis using in vivo luciferase imaging under anesthesia. We observed that after ectopic expression of circZNF609 in cutaneous melanoma A2058 cells, lung metastasis was significantly inhibited (Fig. 4a, c, d) and OS was prolonged compared to that of control cells (Fig. 4b). Additionally, the role of circZNF609 in inhibiting lung metastasis of acral melanoma was also confirmed in the acral melanoma cell line HMY-1. The results of the acral melanoma lung metastasis assay revealed that the number of lung nodules in the circZNF609 overexpression group was less than that in the control group (Fig. 4e-g). To explore the effect of circZNF609 on liver metastasis, we further injected firefly luciferase-expressing HMY-1 cells into the inferior hemispleen of female NOG mice. As expected, the luminescence in livers was weaker and there were fewer liver metastasis nodules in the circZNF609 overexpression group than in the corresponding control group (Fig. 4h-j), demonstrating that ectopic expression of circZNF609 inhibited liver metastasis of acral melanoma. Taken together, these findings suggest that circZNF609 inhibited the metastasis of cutaneous melanoma and acral melanoma in vivo.

### FMRP interacts with circZNF609 in melanoma

**Fig. 5:**
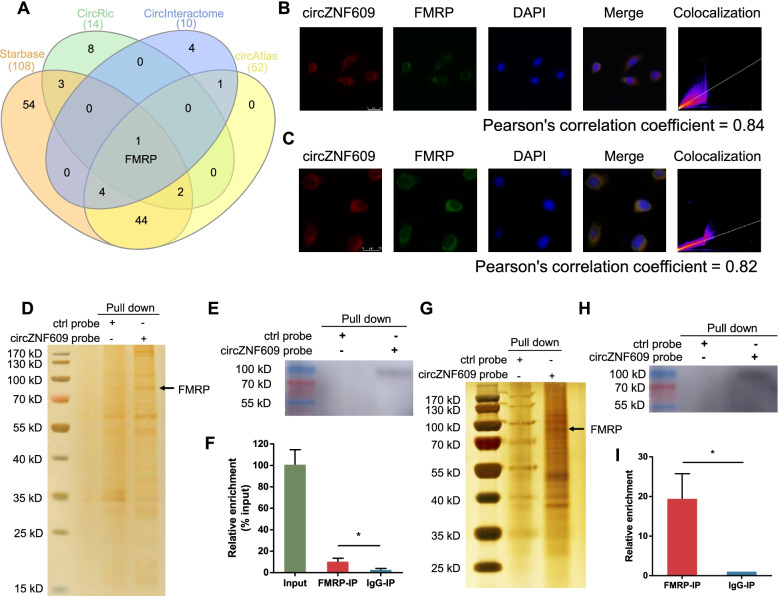
FMRP interacts with circZNF609 in acral melanoma and cutaneous melanoma. **a** Venn diagram showed potential downstream RBP targets of circZNF609 according to bioinformatic analysis results. **b** and **c** The co-localization of circZNF609 and FMRP protein in HMY-1 cells (**b**) and A875 cells (**c**) by immunofluorescence and FISH. **d** Identification of the circZNF609-FMRP complex pulled down by circZNF609 junction probe with protein extracts from HMY-1 cells. **e** Immunoblot analysis of FMRP after pulldown assay with circZNF609 specific junction probe in HMY-1 cells. **f** RIP assays showed the association of FMRP with circZNF609 in HMY-1 cells. **g** Identification of the circZNF609-FMRP complex pulled down by circZNF609 junction probe with protein extracts from A875 cells. **h** Immunoblot analysis of FMRP after pulldown assay with circZNF609 specific junction probe in A875 cells. **i** RIP assays showed the association of FMRP with circZNF609 in A875 cells

Next, we sought to investigate the potential molecular mechanisms of circZNF609 as a metastasis suppressor in melanoma. We first performed bioinformatic analysis to screen circZNF609-interacting proteins (Table S4). Intriguingly, we obtained only 1 candidate circZNF609-interacting protein, FMRP, through overlapping the prediction results of RBP recognition elements in the circZNF609 sequence in starBase [32], CircRic [33], CircInteractome [34] and circAtlas [35] (Fig. 5a). FMRP is an RNA binding protein that regulates a large repertoire of target mRNAs through diverse mechanisms. It has been reported that FMRP levels in breast primary tumors influence metastasis formation [20]. A possible explanation for this finding is that FMRP binds mRNAs involved in epithelial mesenchymal transition (EMT), cytoskeleton remodelling and cell adhesion and regulates their mRNA stability or protein translation. Thus, we hypothesized that circZNF609 might interact with FMRP and modulate its function. By performing IF and FISH assays, we observed colocalization of endogenously expressed circZNF609 and FMRP protein in the cytoplasm of cutaneous melanoma and acral melanoma cells (Fig. 5b, c), suggesting that circZNF609 might associate with FMRP to perform its function. Additionally, we performed RNA pull-down assays, silver staining and western blot analysis in HMY-1 acral melanoma cells (Fig. 5d, e) and A875 cutaneous melanoma cells (Fig. 5g, h), and the results revealed that FMRP is a putative circZNF609-binding protein. Moreover, we observed significant enrichment of circZNF609 in FMRP RIP experiments (Fig. 5f, i). Taken together, these data indicate that FMRP interacts with circZNF609 in cutaneous melanoma and acral melanoma.

### circZNF609 enhances FMRP binding to RAC1 mRNA

**Fig. 6:**
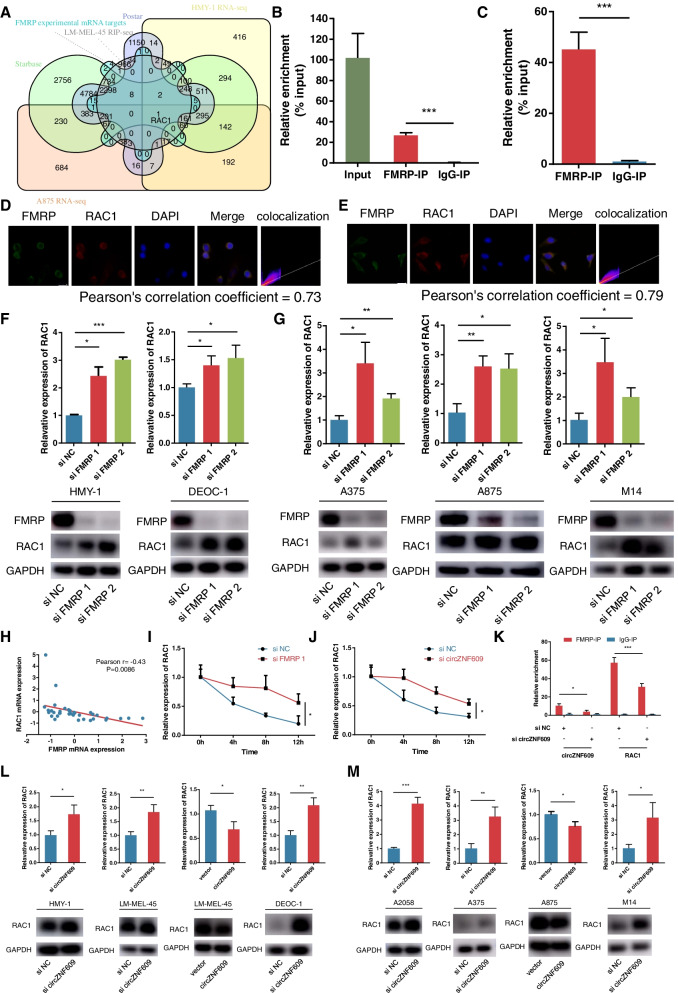
circZNF609 mediates the regulation of RAC1 mRNA stability by binding to FMRP. **a** Venn diagram showed potential downstream mRNA targets of FMRP. **b** and **c** RIP assays showed the association of FMRP with RAC1 mRNA in HMY-1 cells (**b**) and A875 cells (**c**). **d** and **e** The co-localization of FMRP protein and RAC1 mRNA in HMY-1 cells (**d**) and A875 cells (**e**) by immunofluorescence and FISH. **f and g** In acral melanoma cells (**f**) and cutaneous melanoma cells (**g**) transfected with FMRP siRNAs, the levels of RAC1 mRNA (top) were assessed by qRT–PCR analysis, and the levels of RAC1 protein (bottom) were assessed by western blot analysis. **h** The correlation between FMRP mRNA and RAC1 mRNA in Liang’s acral melanoma cohort from the cBioPortal database. **i** and **j** RAC1 mRNA stability was assessed after transfection with FMRP siRNA (**i**) or circZNF609 siRNA (**j**). **k** The expression of circZNF609 and RAC1 mRNA binding to FMRP were assessed after transfection with circZNF609 siRNA. **l** and **m** In acral melanoma cells (**l**) and cutaneous melanoma cells (**m**) transfected with circZNF609 siRNAs or circZNF609 vector, the levels of RAC1 mRNA (top) were assessed by qRT–PCR analysis, and the levels of RAC1 protein (bottom) were assessed by western blot analysis

Because FMRP interacted with circZNF609 in melanoma, we wondered whether circZNF609 modulation regulated FMRP function. Strikingly, silencing of circZNF609 or ectopic expression of circZNF609 in melanoma cells did not alter the expression or cellular distribution of FMRP (Fig. S5a, b). Previous studies have revealed that FMRP plays a critical role in determining the fate of mRNAs in a tissue- and disease-dependent manner, especially in protein translation. In neurons, FMRP is thought to be a translation repressor by integrating with both polysomes and messenger ribonucleoprotein particles (mRNPs) [17]. Although previous studies have mainly focused on the translational regulation of FMRP, there have also been reports showing that FMRP functions as a direct modulator of mRNA stability [14, 36]. Therefore, we performed RNA-seq analyses using circZNF609-depleted cells and control cells and RIP-seq analyses using LM-MEL-45 FMRP-IP RNA and IgG-IP RNA (Fig. 6a). Among these dysregulated mRNAs, we identified RAC1 as the only dysregulated mRNA that met the following strict requirements (Table S5): (1) normalized fold change ≥1.5 in HMY-1 RNA-seq; (2) normalized fold change ≥1.5 in A875 RNA-seq; (3) normalized fold change ≥1.5 in LM-MEL-45 RIP-seq; (4) FMRP mRNA target predicted by the starBase database [32]; (5) FMRP mRNA target predicted by the POSTAR database [37]; and (6) FMRP experimental mRNA target according to a previous study [38]. Then, we performed RIP experiments in acral melanoma and cutaneous melanoma cells. As shown in Fig. 6b and c, RIP assays showed that RAC1 mRNA was enriched in FMRP-IP groups in acral melanoma and cutaneous melanoma cells compared with IgG-IP groups. To further investigate this interaction, we performed FISH and IF in melanoma cells (Fig. 6d, e). Fluorescence results further confirmed the colocalization of FMRP protein and RAC1 mRNA in the cytoplasm of acral melanoma and cutaneous melanoma cells.

Next, we sought to examine the effects of FMRP depletion on RAC1 expression. Strikingly, although previous studies have revealed that FMRP might participate in RAC1 translation in mouse hippocampal neurons and Drosophila [23, 24], our results confirmed that FMRP loss in acral melanoma and cutaneous melanoma could lead to increased RAC1 expression at the mRNA and protein levels (Fig. 6f, g). Moreover, we extracted the transcriptome data of 34 patients with acral melanoma from Liang’s cohort through the cBioPortal database [39, 40] and found a negative correlation between FMRP mRNA and RAC1 mRNA (Fig. 6h), further confirming that FMRP is involved in regulation of RAC1 nontranslational pathways in melanoma. Given that FMRP is known to destabilize mRNA stability, we then assessed RAC1 mRNA stability following FMRP silencing. Using actinomycin D to block de novo transcription, we found that silencing FMRP increased RAC1 mRNA stability (Fig. 6i). Additionally, circZNF609 deficiency or overexpression in acral melanoma (Fig. 6l) and cutaneous melanoma (Fig. 6m) altered RAC1 expression at both the mRNA and protein levels, which was consistent with the RNA-seq results. In line with this finding, we found that circZNF609 deficiency also increased RAC1 mRNA stability (Fig. 6j). We then examined the effects of silencing circZNF609 on the interaction of FMRP and RAC1. RIP analysis indicated that silencing circZNF609 reduced the association of FMRP with circZNF609 but also decreased the binding of FMRP to RAC1 mRNA (Fig. 6k). Collectively, these data demonstrate that circZNF609 affects the abundance of RAC1 by participating in the regulation of RAC1 mRNA stability by FMRP.

### circZNF609 inhibits melanoma metastasis through RAC1

**Fig. 7:**
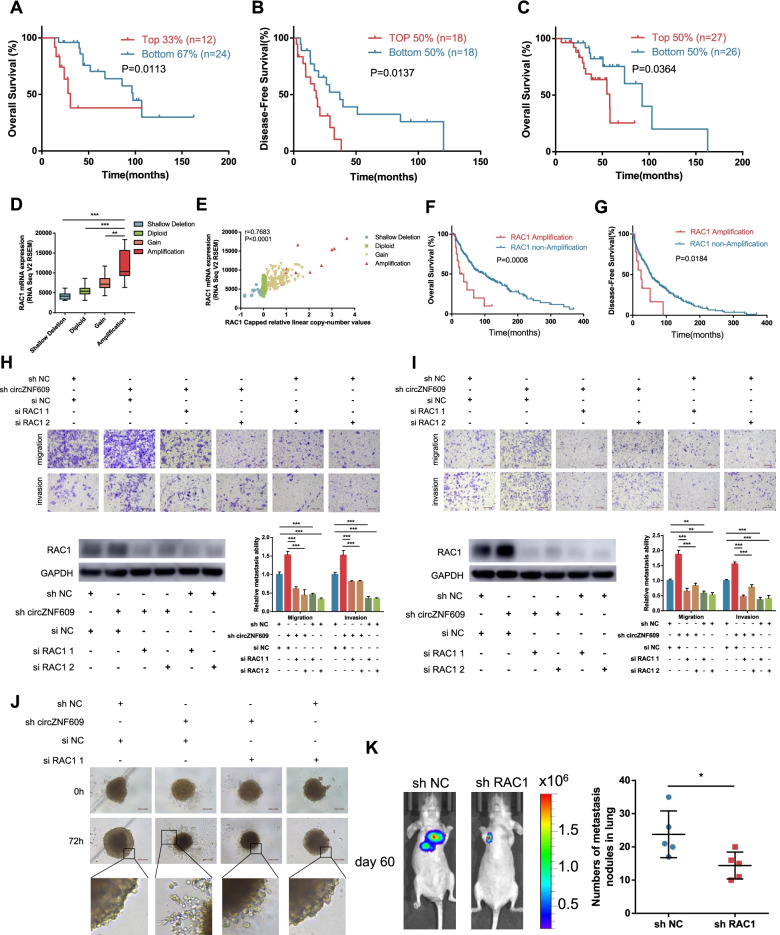
The anti-metastatic effect of circZNF609 is mediated by RAC1. **a** and **b** Kaplan-Meier curves of acral melanoma patients from Liang’s cohort through the cBioPortal database stratified by RAC1 expression for OS (**a**) or DFS (**b**). **c** Kaplan-Meier curves of acral melanoma patients from Beijing Cancer Hospital & Institute stratified by RAC1 expression for OS (*n* = 53). **d** and **e** The association (**d**) and correlation (**e**) between RAC1 mRNA expression and copy-number values from cutaneous melanoma patients through the TCGA database. **f** and **g** Kaplan-Meier curves of cutaneous melanoma patients from TCGA database stratified by RAC1 expression for OS (**f**) or DFS (**g**). **h** and **i** Top, enhanced cell migration and invasion in circZNF609 knockdown HMY-1 cells (**h**) and A875 cells (**i**) was abolished by silencing of RAC1. Bottom, left, the induction of enhanced expression of RAC1 elicited by circZNF609 depletion was abolished by silencing of RAC1. Bottom, right, statistical analysis of Transwell assays. **j** Enhanced cell invasion in circZNF609 knockdown LM-MEL-45 cells was abolished by silencing of RAC1 in 3D spheroid-based Matrigel invasion assay. **k** Representative bioluminescent images (left) and quantification (right) of lung metastatic colonization of nude mice treated with tail-vein injection of firefly luciferase-expressing RAC1-knockdown A2058 cells or control A2058 cells

RAC1 is known to be pro-tumorigenic in numerous cancers [41] (Fig. S6a, b). Our data verified that circZNF609 controls RAC1 mRNA stability by binding with FMRP, thereby regulating the metastasis of melanoma. Therefore, we sought further validation that the circZNF609-FMRP-RAC1 axis governs the metastasis process in acral melanoma and cutaneous melanoma. To this end, we first evaluated the expression levels of RAC1 in melanoma cohorts. From Liang’s cohort in the cBioPortal database, we found that high expression of RAC1 suggested worse OS and disease-free survival (DFS) in acral melanoma patients (Fig. 7a, b). Notably, the high expression levels of RAC1 also predicted worse OS in our acral melanoma cohort (Fig. 7c). Moreover, we found that the expression of RAC1 mRNA was positively correlated with the copy number values of skin cutaneous melanoma patients from TCGA database (Fig. 7d, e), and the group with the highest RAC1 expression, named the RAC1 amplification group, had significantly worse OS and DFS than the nonamplification group (Fig. 7f, g). We then investigated whether the role of circZNF609 in the metastasis of melanomas was dependent on RAC1 modulation. In vitro Transwell and 3D invasion assays demonstrated that silencing RAC1 functionally abolished the induction of melanoma migration and invasion elicited by circZNF609 depletion (Fig. 7h-j). In addition, a lung metastasis model showed that the metastatic nodules in the lungs formed after injection with A2058 cells was reduced by silencing RAC1 (Fig. 7k).

## Discussion

For most cancer types, metastatic spread is the crucial determinant of poor outcomes for cancer patients. According to previous reports, the vast majority of cancer deaths (approximately 90%) are attributable to cancer metastasis rather than primary tumors [42]. In essence, tumorigenesis is a genetic disease, but mutation alone is far from sufficient to explain cancer metastasis processes [43]. In recent years, researchers have gradually discovered that cellular phenotypes regulated by epigenetic mechanisms are critical drivers of cancer metastasis [44].

circRNA is an emerging type of RNA molecule whose role in tumor metastasis is only beginning to be elucidated. CDR1as is downregulated in cutaneous melanoma and regulates the metastatic phenotype of melanoma by interacting with IGF2BP1 instead of binding to miR-7 [30]. Although some circRNAs have been investigated in cutaneous melanoma [30, 45], uveal melanoma [46], oral mucosal melanoma [47] and conjunctival melanoma [48], they have yet to be evaluated in acral melanoma, the most common type of melanoma in Asia.

**Fig. 8:**
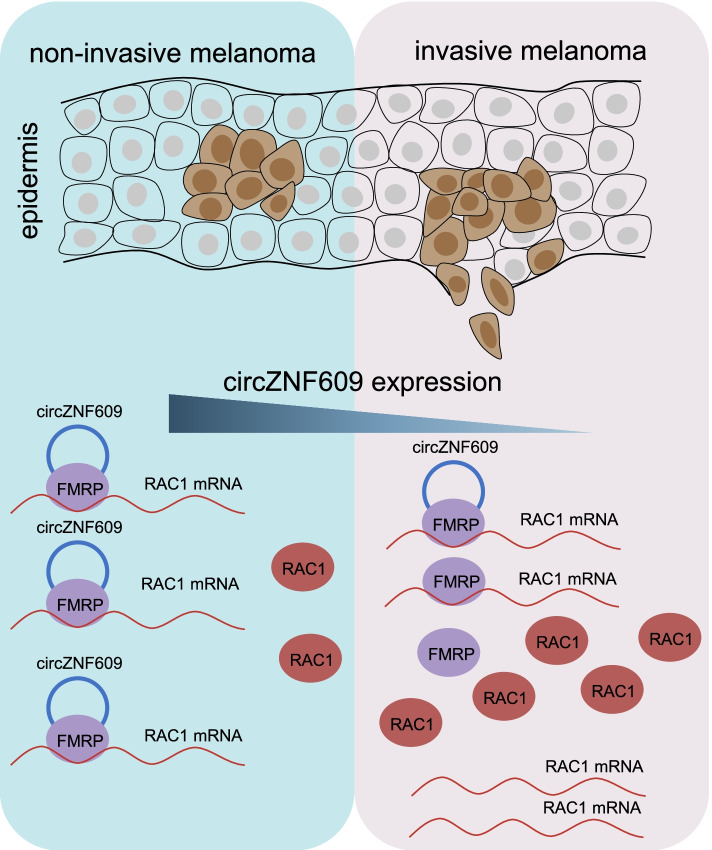
Function and mechanism of circZNF609 during melanoma metastasis. circZNF609 directly binds FMRP and promotes the interaction of FMRP with RAC1 mRNA, thereby inhibiting RAC1 expression and suppressing acral and cutaneous melanoma metastasis

In our study, we found a new mechanism that the circZNF609 can go through the circZNF609/FMRP/RAC1 axis to suppress the melanoma metastasis (Fig. 8). We used repeated Transwell assays to isolate highly invasive sublines from the Asian acral melanoma cell line HMY-1 to study melanoma metastasis, a method that has been used to investigate tumor metastasis in previous studies [49, 50]. We successfully identified a circRNA associated with invasion using microarray data from the cell invasion model. circZNF609 was downregulated in the highly aggressive HMY-1 subline and cutaneous melanoma cells. Loss-of-function and gain-of-function experiments further confirmed that circZNF609 inhibited the migration and invasion of acral and cutaneous melanoma cells in vitro and inhibited melanoma lung metastasis and liver metastasis in vivo, indicating its tumor-suppressive role in melanoma.

circZNF609 was first characterized in 2017 [51]. Legnini et al. demonstrated that circZNF609 participates in the regulation of myoblast proliferation and that circZNF609 can be translated into two polypeptides, although the relationship between the phenotype of circZNF609 and its protein coding ability had not been characterized at that time. In recent years, there have been an increasing number of studies on the ability of circRNAs to encode proteins. However, in our research, we did not observe effects of the two peptides encoded by circZNF609 on the migration and invasion of melanoma cells (data not shown), indicating that the translation ability of circZNF609 is not the key mechanism by which it regulates melanoma metastasis.

Another important mechanism of circRNA is its interaction with RBPs [52–55]. For example, circNSUN2 can regulate the stability of HMGA2 mRNA by forming a circNSUN2/IGF2BP2/HMGA2 RNA-protein ternary complex [56]. In our research, we first discovered through bioinformatics analysis that FMRP protein might bind to circZNF609; colocalization experiments confirmed that both were located in the cytoplasm, further suggesting the possibility of the integration of the two. RNA pull-down experiments and RIP experiments confirmed that circZNF609 bound to FMRP. Interestingly, circZNF609 modulation did not affect the abundance or localization of FMRP in melanoma cells.

Considering that FMRP is a powerful RBP and that FMRP mRNA targets account for 4% or 27% of the transcriptome in different cell types [57–59], we suspect that circZNF609 may affect the function of FMRP as an RBP that regulates the downstream mRNA of FMRP. Previous studies of FMRP have mainly focused on translational regulation, but some studies have found that FMRP can affect mRNA stability. FMRP has been found to regulate the stability of MDM2 mRNA and the long noncoding RNA TUG1 in mouse neural stem cells and mouse neuroblastoma N2a cells [36, 60], respectively. More importantly, FMRP was shown to regulate mouse breast cancer metastasis by modulating the translation and stability of E-cadherin and Vimentin, respectively [20]. Herein, our results strongly suggest that circZNF609 interacts with FMRP to affect the ability of FMRP to regulate the stability of RAC1 mRNA.

RAC1 is a member of the Rho GTPase family and is essential for many normal cellular activities, including phagocytosis, axonal growth, cell adhesion, cell growth and contact inhibition [61]. The RAC1 functions that control cellular adhesion and motility are critical for tumor cells to undergo a multistep metastasis process. Our research revealed that RAC1 was upregulated in a variety of tumors and was related to tumor prognosis. In addition, we analysed RAC1 expression in a skin cutaneous melanoma cohort and an acral melanoma cohort in TCGA and cBioPortal databases and found that high expression of RAC1 indicated a worse clinical prognosis of melanoma patients. A phenotypic rescue experiment further confirmed that circZNF609 plays a role in regulating the metastasis of melanoma through RAC1.

## Conclusion

Our results show that circZNF609 plays a vital role in the metastasis of cutaneous melanoma and acral melanoma through the circZNF609-FMRP-RAC1 axis. Mechanistically, we first discovered that circZNF609 directly binds FMRP and promotes the interaction of FMRP with RAC1 mRNA, thereby inhibiting RAC1 mRNA and protein levels and suppressing melanoma metastasis. These findings provide an important theoretical basis for further elucidating the pathogenesis of cutaneous and acral melanoma and developing targeted therapy.

## Supplementary Information

**Additional file 1: Fig. S1.** Verification of highly invasive HMY-1 cell sublines. **a** The growth pattern of HMY-1 and highly invasive HMY-1 cell sublines. **b** and **c** In vitro Transwell assay to verify the different metastatic potential between HMY-1 and highly invasive HMY-1 sublines. **d** The expression of circZNF609 in HMY-1 and highly invasive HMY-1 cell sublines. **Fig. S2.** Abundant circRNAs in human melanomas. **a** and **b** The abundant circRNAs in 3 cutaneous melanoma cell lines (**a**) and 4 normal skin tissues(**b**). **c** circRNAs riched in melanoma cell lines and normal skin tissues. **d** Selection of circRNAs that may truly participate in the progression of melanoma. **Fig. S3.** circZNF609 knockdown and overexpression efficiency in melanoma. **a-c** circZNF609 knockdown efficiency (left) and overexpression efficiency (right) in HMY-1 (**a**), A2058 (**b**) and A875 (**c**). **Fig. S4.** Cell migratory and invasive capabilities were assessed after modulation of circZNF609. **a** and **b** circZNF609 overexpression in 2 acral melanoma cells (**a**) and 2 cutaneous melanoma cells (**b**) suppressed cell migration and invasion. **c** Cell migratory capability was assessed by wound healing assay after knocking down or overexpressing circZNF609 in 2 melanoma cells. **Fig. S5.** The expression and cellular distribution of FMRP were assessed after circZNF609 modulation. **a** and **b** circZNF609 modulation in melanoma cells did not alter the expression (**a**) or cellular distribution (**b**) of FMRP. **Fig. S6.** RAC1 expression and survival analysis in cancers. **a** The high expression of RAC1 in numerous cancers. **b** The Kaplan-Meier survival analysis of RAC1 in numerous cancers. **Fig. S7.** Unprocessed figures of western blot assays. **Fig. S8.** Unprocessed figures of western blot assays.

**Additional file 2: Table S1.** The detailed information of human melanoma cell lines used in this study. **Table S2.** The sequences of primers and oligonucleotides used in this study. **Table S3.** circRNAs abundant in 3 cutaneous melanoma cell lines and 4 normal skin tissues. **Table S4.** Prediction and selection of circZNF609-associated RBPs. **Table S5.** Selection of FMRP targets in acral melanoma and cutaneous melanoma.

## Data Availability

The datasets used and/or analyzed during the current study are available from the corresponding author on reasonable request.

## Abbreviations

circRNA: Circular RNA
FMRP: Fragile X mental retardation protein
FXS: Fragile X Syndrome
RAC1: RAS-related C3 botulinum toxin substrate 1
ATCC: American Type Culture Collection
JCRB: Japanese Cancer Research Resources Bank
IHC: Immunohistochemistry
qRT–PCR: Quantitative reverse transcription polymerase chain reaction
FISH: Fluorescence in situ hybridization
IF: Immunofluorescence
H&E: Haematoxylin and eosin
RIP: RNA immunoprecipitation

## References

[1] Budman DR, Camacho E, Wittes RE (1978). The current causes of death in patients with malignant melanoma. Eur J Cancer.

[2] Davies MA (2011). Prognostic factors for survival in melanoma patients with brain metastases. Cancer.

[3] Patel JK (1978). Metastatic pattern of malignant melanoma. A study of 216 autopsy cases. Am J Surg.

[4] Hayward NK (2017). Whole-genome landscapes of major melanoma subtypes. Nature.

[5] Schadendorf D (2018). Melanoma. Lancet.

[6] Bai X (2017). MAPK pathway and TERT promoter gene mutation pattern and its prognostic value in melanoma patients: a retrospective study of 2,793 cases. Clin Cancer Res.

[7] Harland R, Misher L (1988). Stability of RNA in developing Xenopus embryos and identification of a destabilizing sequence in TFIIIA messenger RNA. Development.

[8] Pamudurti NR (2017). Translation of CircRNAs. Mol Cell.

[9] Xia P (2018). A circular RNA protects dormant hematopoietic stem cells from DNA sensor cGAS-mediated exhaustion. Immunity.

[10] Hollensen AK (2020). circZNF827 nucleates a transcription inhibitory complex to balance neuronal differentiation. Elife.

[11] Liu CX (2019). Structure and degradation of circular RNAs regulate PKR activation in innate immunity. Cell.

[12] Zheng Q (2016). Circular RNA profiling reveals an abundant circHIPK3 that regulates cell growth by sponging multiple miRNAs. Nat Commun.

[13] Pfeiffer BE, Huber KM (2009). The state of synapses in fragile X syndrome. Neuroscientist.

[14] Zalfa F (2007). A new function for the fragile X mental retardation protein in regulation of PSD-95 mRNA stability. Nat Neurosci.

[15] Zhang W (2014). A feed-forward mechanism involving Drosophila fragile X mental retardation protein triggers a replication stress-induced DNA damage response. Hum Mol Genet.

[16] Bechara EG (2009). A novel function for fragile X mental retardation protein in translational activation. PLoS Biol.

[17] Laggerbauer B (2001). Evidence that fragile X mental retardation protein is a negative regulator of translation. Hum Mol Genet.

[18] Mazroui R (2002). Trapping of messenger RNA by fragile X mental retardation protein into cytoplasmic granules induces translation repression. Hum Mol Genet.

[19] Schultz-Pedersen S (2001). Evidence of decreased risk of cancer in individuals with fragile X. Am J Med Genet.

[20] Luca R (2013). The fragile X protein binds mRNAs involved in cancer progression and modulates metastasis formation. EMBO Mol Med.

[21] Shen Z (2021). FMRP regulates STAT3 mRNA localization to cellular protrusions and local translation to promote hepatocellular carcinoma metastasis. Commun Biol.

[22] Bid HK (2013). RAC1: an emerging therapeutic option for targeting cancer angiogenesis and metastasis. Mol Cancer Ther.

[23] Lee A (2003). Control of dendritic development by the Drosophila fragile X-related gene involves the small GTPase Rac1. Development.

[24] Majumder P (2016). Co-regulation of mRNA translation by TDP-43 and fragile X syndrome protein FMRP. Acta Neuropathol.

[25] Vinci M, Box C, Eccles SA (2015). Three-dimensional (3D) tumor spheroid invasion assay. J Vis Exp.

[26] Vinci M (2012). Advances in establishment and analysis of three-dimensional tumor spheroid-based functional assays for target validation and drug evaluation. BMC Biol.

[27] Ivascu A, Kubbies M (2006). Rapid generation of single-tumor spheroids for high-throughput cell function and toxicity analysis. J Biomol Screen.

[28] Xu C, Zhang J (2021). Mammalian circular RNAs result largely from splicing errors. Cell Rep.

[29] Xia S (2018). CSCD: a database for cancer-specific circular RNAs. Nucleic Acids Res.

[30] Hanniford D (2020). Epigenetic silencing of CDR1as drives IGF2BP3-mediated melanoma invasion and metastasis. Cancer Cell.

[31] Glazar P, Papavasileiou P, Rajewsky N (2014). circBase: a database for circular RNAs. RNA.

[32] Li JH (2014). starBase v2.0: decoding miRNA-ceRNA, miRNA-ncRNA and protein-RNA interaction networks from large-scale CLIP-Seq data. Nucleic Acids Res.

[33] Ruan H (2019). Comprehensive characterization of circular RNAs in ~ 1000 human cancer cell lines. Genome Med.

[34] Dudekula DB (2016). CircInteractome: a web tool for exploring circular RNAs and their interacting proteins and microRNAs. RNA Biol.

[35] Wu W, Ji P, Zhao F (2020). CircAtlas: an integrated resource of one million highly accurate circular RNAs from 1070 vertebrate transcriptomes. Genome Biol.

[36] Li Y (2016). MDM2 inhibition rescues neurogenic and cognitive deficits in a mouse model of fragile X syndrome. Sci Transl Med.

[37] Hu B (2017). POSTAR: a platform for exploring post-transcriptional regulation coordinated by RNA-binding proteins. Nucleic Acids Res.

[38] Pasciuto E, Bagni C (2014). SnapShot: FMRP mRNA targets and diseases. Cell.

[39] Cerami E (2012). The cBio cancer genomics portal: an open platform for exploring multidimensional cancer genomics data. Cancer Discov.

[40] Liang WS (2017). Integrated genomic analyses reveal frequent TERT aberrations in acral melanoma. Genome Res.

[41] Tang Z (2017). GEPIA: a web server for cancer and normal gene expression profiling and interactive analyses. Nucleic Acids Res.

[42] Lambert AW, Pattabiraman DR, Weinberg RA (2017). Emerging biological principles of metastasis. Cell.

[43] Vogelstein B (2013). Cancer genome landscapes. Science.

[44] Chatterjee A, Rodger EJ, Eccles MR (2018). Epigenetic drivers of tumourigenesis and cancer metastasis. Semin Cancer Biol.

[45] Wei CY (2020). Circular RNA circ_0020710 drives tumor progression and immune evasion by regulating the miR-370-3p/CXCL12 axis in melanoma. Mol Cancer.

[46] Yang X (2018). Novel circular RNA expression profile of uveal melanoma revealed by microarray. Chin J Cancer Res.

[47] Ju H (2018). Altered expression pattern of circular RNAs in metastatic oral mucosal melanoma. Am J Cancer Res.

[48] Shang Q (2019). Altered expression profile of circular RNAs in conjunctival melanoma. Epigenomics.

[49] Liu H (2018). Invasion-related circular RNA circFNDC3B inhibits bladder cancer progression through the miR-1178-3p/G3BP2/SRC/FAK axis. Mol Cancer.

[50] Han K (2020). CircLONP2 enhances colorectal carcinoma invasion and metastasis through modulating the maturation and exosomal dissemination of microRNA-17. Mol Cancer.

[51] Legnini I (2017). Circ-ZNF609 is a circular RNA that can be translated and functions in Myogenesis. Mol Cell.

[52] Tsitsipatis D (2021). AUF1 ligand circPCNX reduces cell proliferation by competing with p21 mRNA to increase p21 production. Nucleic Acids Res.

[53] Guarnerio J (2019). Intragenic antagonistic roles of protein and circRNA in tumorigenesis. Cell Res.

[54] Luo J (2019). Guidance of circular RNAs to proteins' behavior as binding partners. Cell Mol Life Sci.

[55] Shang Q (2019). The novel roles of circRNAs in human cancer. Mol Cancer.

[56] Chen RX (2019). N(6)-methyladenosine modification of circNSUN2 facilitates cytoplasmic export and stabilizes HMGA2 to promote colorectal liver metastasis. Nat Commun.

[57] Ashley CT (1993). Human and murine FMR-1: alternative splicing and translational initiation downstream of the CGG-repeat. Nat Genet.

[58] Brown V (2001). Microarray identification of FMRP-associated brain mRNAs and altered mRNA translational profiles in fragile X syndrome. Cell.

[59] Darnell JC (2011). FMRP stalls ribosomal translocation on mRNAs linked to synaptic function and autism. Cell.

[60] Guo Y (2018). Interplay between FMRP and lncRNA TUG1 regulates axonal development through mediating SnoN-Ccd1 pathway. Hum Mol Genet.

[61] Heasman SJ, Ridley AJ (2008). Mammalian rho GTPases: new insights into their functions from in vivo studies. Nat Rev Mol Cell Biol.

